# Identification of non-activated lymphocytes using three-dimensional refractive index tomography and machine learning

**DOI:** 10.1038/s41598-017-06311-y

**Published:** 2017-07-27

**Authors:** Jonghee Yoon, YoungJu Jo, Min-hyeok Kim, Kyoohyun Kim, SangYun Lee, Suk-Jo Kang, YongKeun Park

**Affiliations:** 1 Department of Physics, Korea Advanced Institute of Science and Technology (KAIST), Daejeon, 34141 Republic of Korea; 2 KAIST Institute Health Science and Technology, Daejeon, 34141 Republic of Korea; 3 Department of Biological Sciences, KAIST, Daejeon, 34141 Republic of Korea; 4 Tomocube, Inc., Daejeon, 34051 Republic of Korea; 5 Present Address: Department of Physics, University of Cambridge, Cambridge, CB3 0HE UK

## Abstract

Identification of lymphocyte cell types are crucial for understanding their pathophysiological roles in human diseases. Current methods for discriminating lymphocyte cell types primarily rely on labelling techniques with magnetic beads or fluorescence agents, which take time and have costs for sample preparation and may also have a potential risk of altering cellular functions. Here, we present the identification of non-activated lymphocyte cell types at the single-cell level using refractive index (RI) tomography and machine learning. From the measurements of three-dimensional RI maps of individual lymphocytes, the morphological and biochemical properties of the cells are quantitatively retrieved. To construct cell type classification models, various statistical classification algorithms are compared, and the *k*-NN (*k* = 4) algorithm was selected. The algorithm combines multiple quantitative characteristics of the lymphocyte to construct the cell type classifiers. After optimizing the feature sets via cross-validation, the trained classifiers enable identification of three lymphocyte cell types (B, CD4+ T, and CD8+ T cells) with high sensitivity and specificity. The present method, which combines RI tomography and machine learning for the first time to our knowledge, could be a versatile tool for investigating the pathophysiological roles of lymphocytes in various diseases including cancers, autoimmune diseases, and virus infections.

## Introduction

Lymphocytes consist of various cell types including B, helper (CD4+) T, cytotoxic (CD8+) T, and regulatory T cells, and play crucial roles in the adaptive immune system^1^. Each lymphocyte cell type has different functions: B lymphocytes produce antibodies, and T lymphocytes recognize a specific antigen and execute effector functions. The lymphocyte population and function are tightly regulated to defend the host against harmful invaders or abnormal conditions^1, 2^. Disturbances in lymphocyte function and regulation are related to various diseases including cancers^3–5^, autoimmune diseases^6, 7^, and virus infections^8, 9^.

To understand the roles of different types of lymphocytes, several methods based on labelling techniques have been developed to identify and separate lymphocyte cell types. Because different types of non-activated lymphocytes have very similar cellular morphology such as a large nucleus with small cytosolic regions and round shapes, it is impossible to discriminate lymphocyte cell types with conventional optical methods such as bright-field microscopy or phase contrast microscopy^10^. To overcome this difficulty, specific surface membrane proteins, known as surface markers, are recognized and tagged with magnetic beads or fluorescence molecules via antigen-antibody binding. Then each type of lymphocytes can be distinguished and separated by magnetic forces or fluorescence signals^11^. Targeting surface markers is a precise and efficient approach to determine the cell types; however, labelling methods have potential risks of altering cellular functions by modifying membrane protein structures. In addition, labelling methods have limitations in the number of cell types that can be identified simultaneously due to the limited multiplexing capability of the labelling agents^12^.

Label-free approaches such as mass spectroscopy^12^ and Raman spectroscopy^10^ have also been introduced to overcome the limitations of labelling methods because these spectroscopic methods exploit intrinsic biochemical properties of cells. Mass spectroscopy measures cellular biochemical properties which enable the profiling of lymphocyte proteins as well as the identification of lymphocyte cell types. However, it has a limitation in live-cell analysis due to the homogenization process of the cells. Raman spectroscopy measures molecular vibrations and also characterizes biochemical properties of a sample. Raman spectroscopy permits label-free live-cell analysis of lymphocytes with high accuracy; however, it requires a bulky optical system and long acquisition time (typically several seconds per cell) which limits its practical use.

Here, we present a method to identify lymphocyte cell types by exploiting optical diffraction tomography (ODT) and machine learning. ODT is a label-free imaging technique that measures a three-dimensional (3-D) refractive index (RI) tomogram of a sample which provides quantitative morphological and biochemical information^13, 14^. ODT has been widely used to study various biological samples including red blood cells^15–22^, white blood cells (WBC)^23, 24^, hepatocytes^25^, cancer cells^16, 26–32^, neurons^32, 33^, bacteria^34–36^, phytoplankton^37^, and hair^38^. In our previous study, we reported that ODT enables the quantitative analysis of WBCs including lymphocytes and macrophages^23^; we demonstrated that the two WBC subtypes could be discriminated using ODT. However, we were unable to simply identify lymphocyte cell types due to their nearly indistinguishable cellular morphology and biochemical characteristics.

In the present study, we use machine learning techniques to systematically interrogate the subtle differences between the lymphocyte cell types. Since RI is an intrinsic property of each biochemical component, the measured 3-D RI tomograms should encode the cell-type-specific fingerprints. However, it is difficult to manually discover such fingerprint information due to the complexity of 3-D tomograms. To solve this difficulty, statistical classification methods construct classification models by combining multiple features in a data-driven manner, instead of conventional hypothesis-driven investigations. This approach is especially powerful for high-dimensional data that are extremely difficult to be manually processed by humans due to the complexity and large size^39^, and thus machine learning techniques have been widely used to solve complex biological problems: identification of bacterial species^40, 41^, discrimination of WBC subtypes^42, 43^, investigation of pathophysiological conditions^44–46^, and classification of kinetic cell states^47^. Here we combine 3-D RI tomography and machine learning for the first time; we exploit statistical classification techniques to establish the cell type classifiers using the quantitative morphological and biochemical information extracted from the 3-D RI tomograms of individual lymphocytes. The trained classifiers enable identification of three lymphocyte cell types (B, CD4+ T, and CD8+ T cells) with high sensitivity and specificity.

## Results

**Figure 1:**
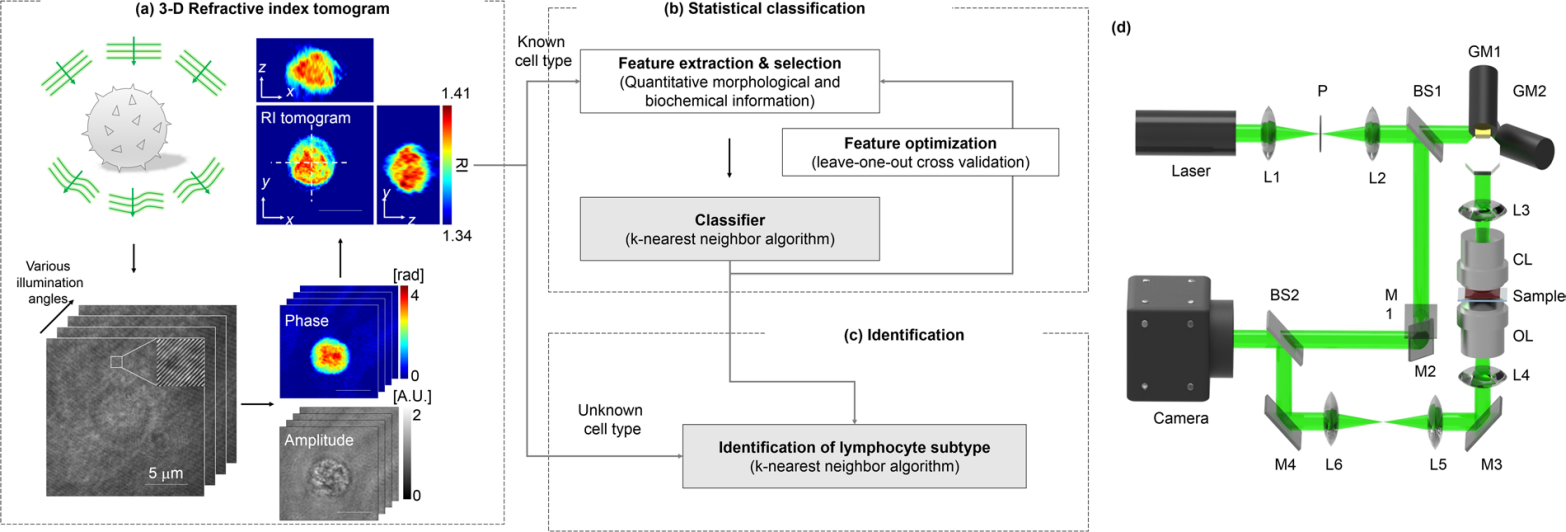
Schematic diagrams of the label-free identification of individual lymphocytes using optical diffraction tomography and machine learning. (**a**) Procedures for label-free measurement of 3-D RI tomograms of lymphocytes. Multiple holograms of a lymphocyte are measured by changing the angle of illumination. Optical field information with various incident angles was retrieved from the measured holograms, and then 3-D RI tomograms were reconstructed using an optical diffraction tomography algorithm. Scale bar, 5 μm. (**b**) Training a statistical cell type classifier for identifying lymphocyte cell types using the *k*-NN algorithm. The multiple quantitative morphological and biochemical features of individual lymphocytes were combined to recognize and exploit the cell-type-specific fingerprints via supervise learning. (**c**) Identifying the cell types of newly observed individual lymphocytes with the established classifier. (**d**) Schematic of the experimental setup. A Mach-Zehnder interferometric microscope equipped with a 2-D scanning galvanomirror (GM) was used for measuring the holograms of individual lymphocytes. L1−6, lenses; P, pinhole; BS1−2, beam splitters; M1−4, mirrors; CL, condenser lens; OL, objective lens.

The overall procedures for identification of non-activated lymphocytes are summarized in Fig. 1. The present approach involves three steps: (i) measurement of the 3-D RI tomograms of individual lymphocytes (Fig. 1a), (ii) construction of the statistical cell type classifiers using the quantitative biochemical and morphological features extracted from the tomograms (Fig. 1b), and (iii) identification of the new individual lymphocytes using the established classifiers (Fig. 1c).

Figure 1a shows the procedures for reconstructing the RI tomograms of the individual lymphocytes. To reconstruct a 3-D RI tomograms, multiple 2-D holograms of a cell are measured at various angles of illuminations using an interferometric microscope^48^ (Fig. 1d). A coherent laser beam is split into two arms by a beam splitter. One arm passes through a sample, and then the diffracted light from the sample is projected onto a camera plane through a microscope. At the camera plane, the sample beam interferes with the other arm to generate a spatially modulated hologram. The angle of the beam impinging onto the sample is controlled by a dual-axis galvanomirror. From the measured holograms, complex optical fields consisting of both the amplitude and quantitative phase images are retrieved using a field retrieval algorithm^49, 50^. Then, a 3-D RI tomogram of a lymphocyte is reconstructed using the multiple optical amplitude and phase information via an optical diffraction tomography algorithm^14, 51^ (see *Methods*).

**Figure 2:**
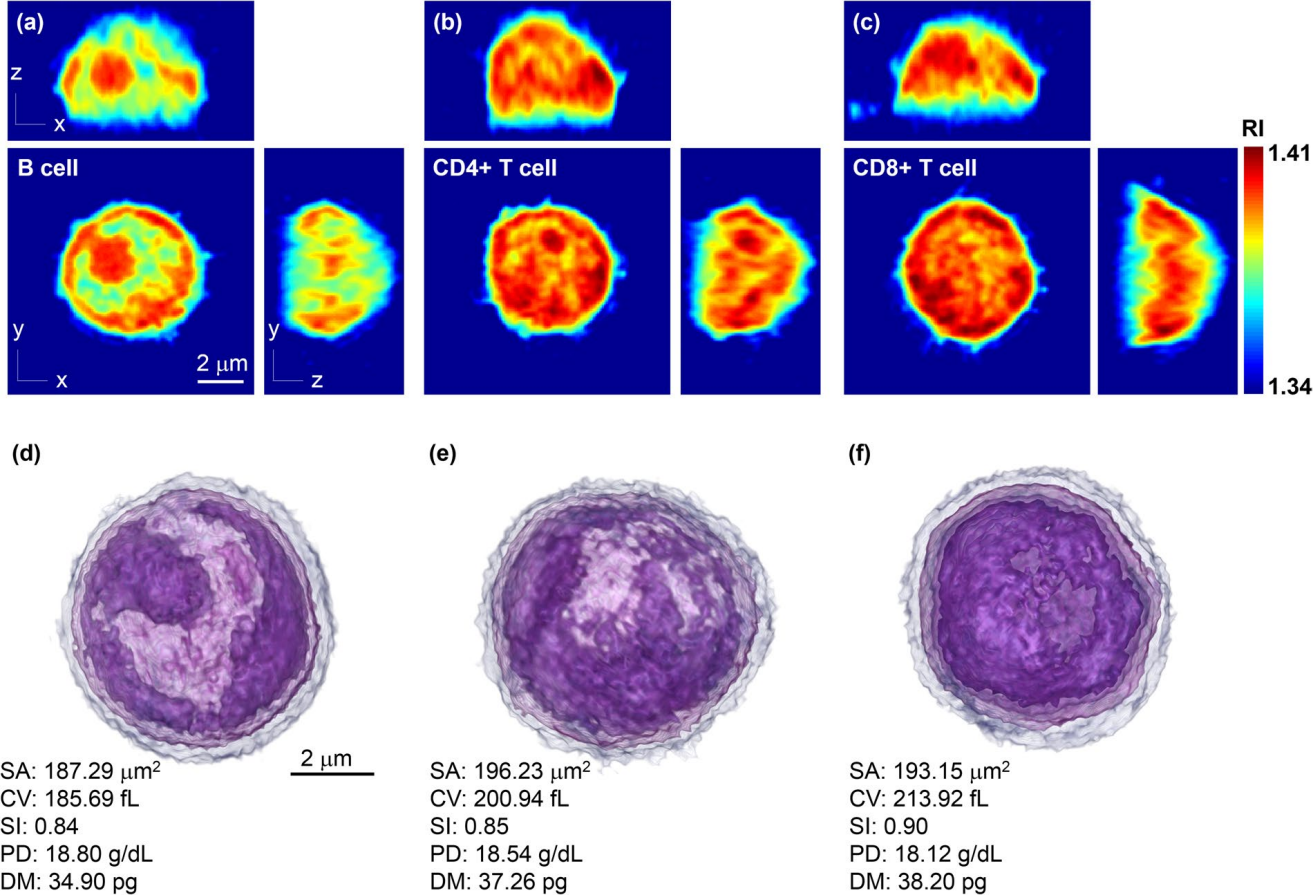
Representative 3-D RI tomograms of each lymphocyte cell type and the 3-D rendered images with quantitative characterization. Cross-sectional slices of a RI tomogram of (**a**) B cell, (**b**) CD4+ T cell, and (**c**) CD8+ T cell. Scale bar, 2 μm. (**d**–**f**) 3-D rendered tomograms and quantitative characterization of the morphological and biochemical features of (**a**–**c**). Scale bar, 2 μm. SA, surface area; CV, cellular volume; SI, sphericity; PD, protein density; DM, dry mass.

We obtained and sorted three lymphocyte cell types, B, CD4+ T, and CD8+ T cells, from mice peripheral blood (see *Methods*). The measured 3-D RI tomograms of the individual lymphocytes are shown in Fig. 2. The cross-sectional slices of the representative 3-D tomograms of the three cell types are shown in Fig. 2a–c, respectively. To facilitate visualization, the tomographic data was 3-D rendered using a customized transfer function in a commercialized software (TomoStudio^TM^, Tomocube Inc., Republic of Korea) to resemble haematoxylin and eosin staining (Fig. 2d–f and Supplementary Videos 1–3). Clearly, the measured RI distribution visualizes the cellular boundaries and internal organelles such as nuclear membranes and nucleoli. The B cell shows a well-defined nucleus and nucleoli with RI values ranging from 1.34 to 1.41. We also note that the RI values of the cytosolic regions of the CD4+ and CD8+ T cells are higher than that of the B cell. Despite of these slight differences in RI distribution, the cell-type-specific fingerprints for cell type identification could not be clearly defined through visual interrogation, mainly due to the cell-to-cell variations.

**Figure 3:**
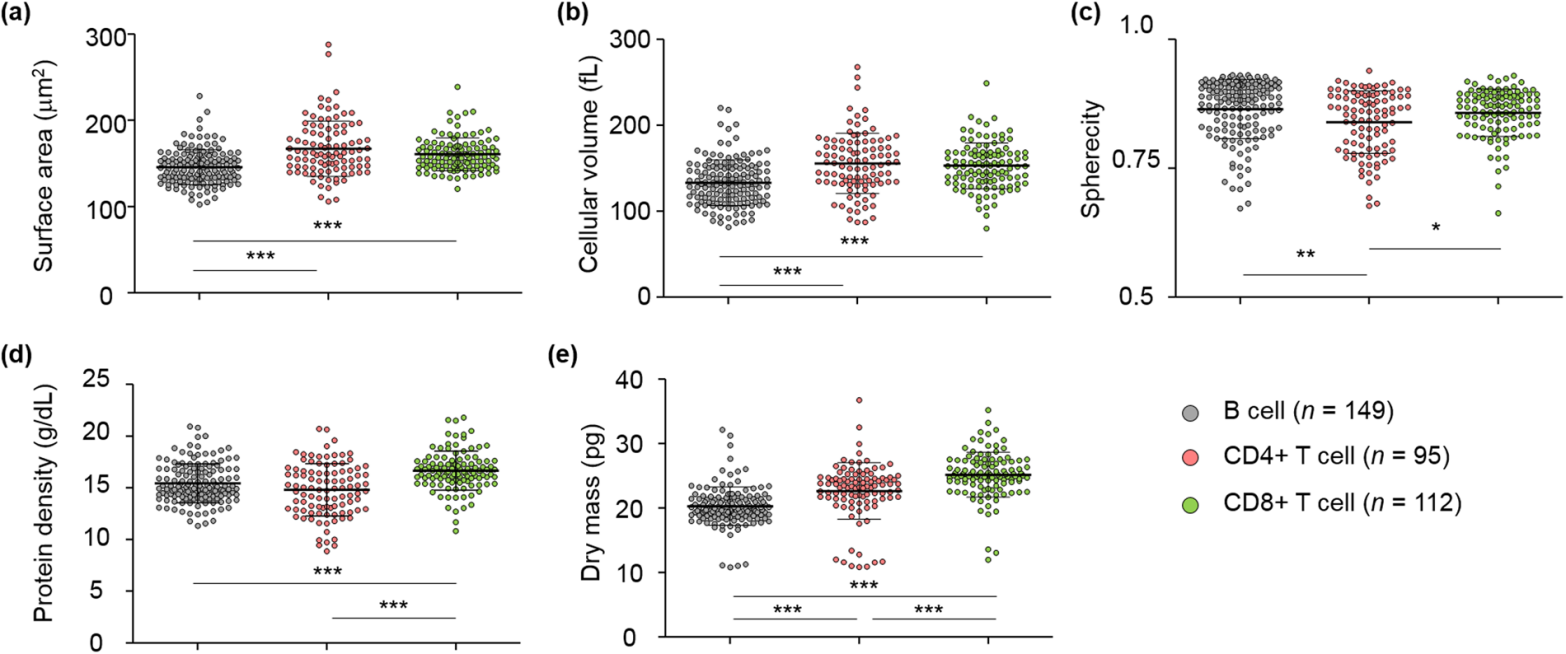
Quantitative analysis of the morphological and biochemical parameters of the individual B, CD4+ T, and CD8+ T cells. (**a**–**c**) The scatter plots of the morphological features: (**a**) surface area, (**b**) cell volume, and (**c**) sphericity. (**d**,**e**) The scatter plots of the biochemical features: (**d**) protein density and (**e**) dry mass. Each symbol indicates a single-cell measurement. Note that a single 3-D RI tomogram simultaneously provides all five parameters. Horizontal black lines, mean values; vertical lines with intervals, standard deviation. *P < 0.05; **P < 0.01; ***P < 0.001.

Next we extracted the quantitative characteristics of the individual lymphocytes from the 3-D RI tomograms as illustrated in Fig. 3 (n = 149, 95, and 112 for B, CD4+ T, and CD8+ T cells, repsectively). The five quantitative morphological (surface area, volume, and sphericity) and biochemical (protein density and dry mass) parameters were calculated from the tomograms (see *Methods*). The cellular surface area and volume of the lymphocytes were simply calculated from the segmented (RI threshold = 1.340) voxel information of the 3-D tomograms, and then the sphericity, a dimensionless parameter that indicates the roundness of the cellular morphology, was obtained by the ratio of the surface area and volume. The cellular protein density and dry mass were retrieved using the RI values that are linearly proportional to the local concentration of non-aqueous molecules (mostly proteins).

To investigate the differences among the lymphocyte cell types, a statistical analysis was conducted on the quantitative parameters of the three lymphocyte cell types. First we studied the inter-type differences in the quantitative morphological features. The cellular surface areas of the B, CD4+ T, and CD8+ T lymphocytes were 145.87 ± 20.25, 167.23 ± 32.08, and 160.80 ± 19.12 μm^2^, respectively (Fig. 3a). The B cells had significantly smaller cellular surface areas compared to the T cells (*P* < 0.001), while there was no significant difference between the CD4+ and CD8+ T cells. The cellular volumes of the lymphocytes also show a similar tendency with the result of the cellular surface area analysis. The cellular volumes of the B cells (133.43 ± 26.47 fL) were significantly smaller (*P* < 0.001) compared to those of the CD4+ T (155.73 ± 35.14 fL) and CD8+ T (152.77 ± 26.52 fL) cells (Fig. 3b), while the CD4+ and CD8+ T cells had similar cellular volumes. The sphericities were 0.86 ± 0.06, 0.84 ± 0.06, and 0.86 ± 0.05 for the B, CD4+ T, and CD8+ T cells, respectively (Fig. 3c). The sphericities of the CD4+ T cells were statistically smaller than those of the B cells (*P* < 0.01) and CD8+ T cells (*P* < 0.05). Note that all the lymphocyte cell types had high sphericity values which suggest the round shapes of the lymphocytes.

We also compared the biochemical properties of the lymphocyte cell types. The cellular protein densities of the B, CD4+ T, and CD8+ T cells were 15.43 ± 1.88, 14.81 ± 2.54, and 16.66 ± 1.88 g/dL, respectively (Fig. 3d). The CD8+ T cells had significantly higher cellular protein densities compared to those of the others (*P* < 0.001). The cellular dry mass was 20.28 ± 2.97, 22.65 ± 4.49, and 25.19 ± 3.51 pg for the B, CD4+ T, and CD8+ T cells, respectively, and showed significant differences (*P* < 0.001) between all the cell types (Fig. 3e). The B cells had a smaller cellular dry mass compared to the T cells. Moreover, the CD8+ T cells were statistically heavier than the CD4+ T cells. To summarize, we observed several statistical differences in both morphological and biochemical features at the *population* level. However, we failed to manually establish cell type classifiers based on these parameters at the *single-cell* level, due to the high dimensionality of the feature space and the cell-to-cell variations.

To achieve accurate identification of individual lymphocytes, we employed a machine learning approach to combine and exploit multiple features encoded in the 3-D RI tomograms. First we enlarged the feature space by extracting the quantitative parameters calculated at 20 different threshold RI values (from 1.340 to 1.378 with an increment of 0.002) to reveal the information specific to intracellular components in addition to the overall morphology (Supplementary Fig. 1). Then we systematically investigated the 100-dimensional feature space (5 parameters per threshold value), which is impractical be manually explored, using statistical classification models. We employed the well-known *k*-nearest neighbours (*k*-NN) algorithm^52, 53^ (see *Methods*) with *k* = 4 after comparing several models (Supplementary Fig. 2).

The statistical classification, or supervised machine learning, was performed through the training and test stages as explained earlier (Fig. 1b,c). We randomly split each lymphocyte subtype data into 70% and 30% of training (*n* = 104, 66, and 77 for B, CD4+ T, and CD8+ T cells, repsectively) and test sets (*n* = 45, 29, and 35 for B, CD4+ T, and CD8+ T cells, repsectively), respectively. First we constructed the cell type classifiers by combining subsets of the features extracted from the training data set. We exhaustively searched the optical combinations of the features via cross-validation (see *Methods*) and the classifier with the best training accuracy was selected. The established classifiers exploit the cell-type-specific fingerprints recognized from the high-dimensional feature space. To test if these fingerprints are general to new lymphocytes, we identified the individual lymphocytes categorized as the test data. The test accuracy and its sub-parameters, called sensitivity (true positive results over all positive inputs) and specificity (true negative results over all negative inputs), were calculated by comparing the machine-predicted and true cell types.

**Figure 4:**
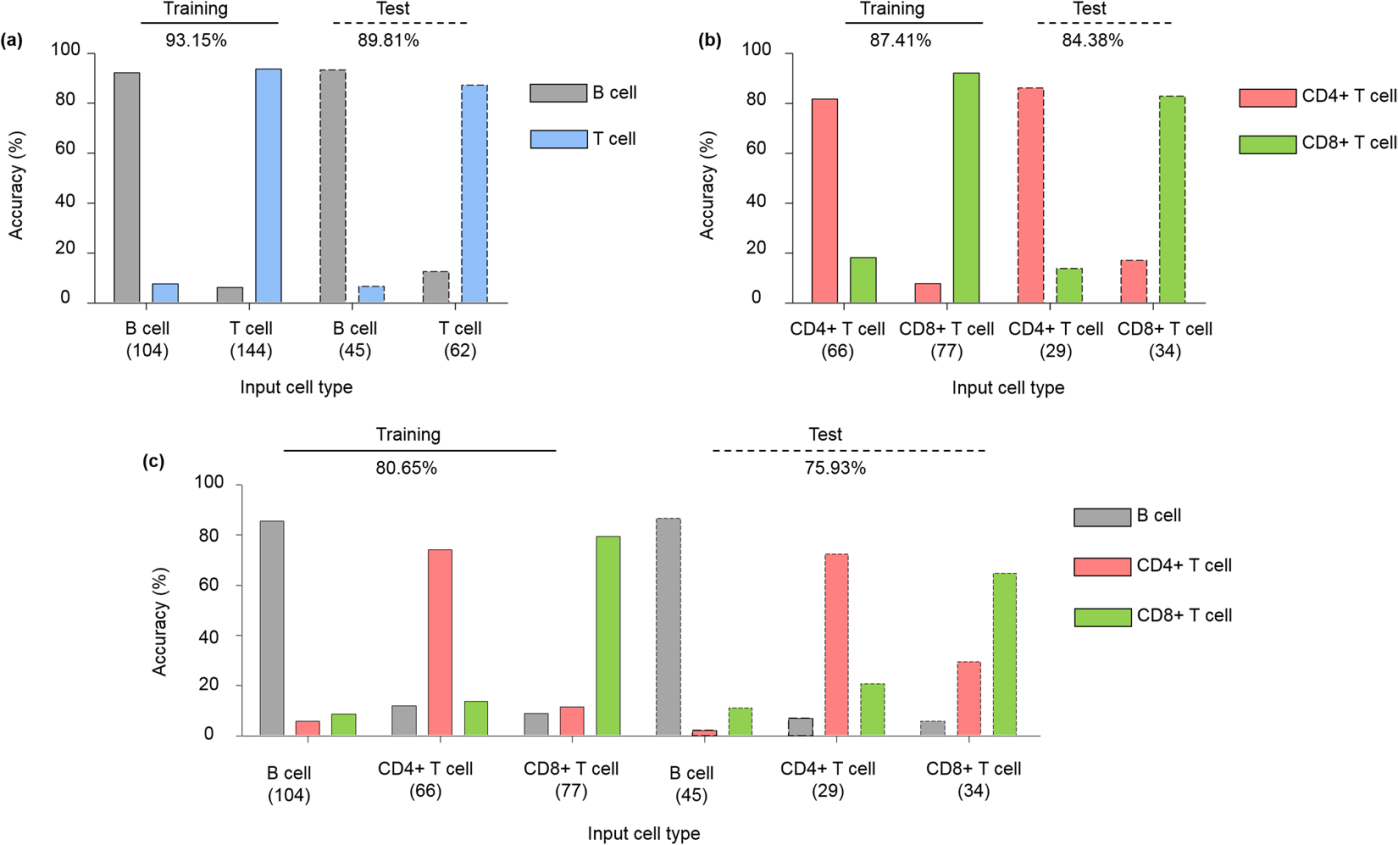
Identification performance of the optimized lymphocyte cell type classifiers. The performance of cell type identification was illustrated for (**a**) binary classification of B and T cells, (**b**) binary classification of CD4+ and CD8+ T cells, and (**c**) multiclass classification of all three lymphocyte cell types, for both training and test sets. Note the small difference between the training and test cases, suggesting nice generalization of the trained classifiers. The numbers below the name of each cell type indicate the number of cells used.

Figure 4 and Tables 1–3 illustrate the identification performance for both training and test stages. We performed statistical classification on three different combinations of the lymphocyte cell types: (i) binary classification of B and T lymphocytes, (ii) binary classification of the two T lymphocyte types (CD4+ and CD8+), and (iii) multiclass classification of all three types of lymphocytes. First, the two T cell types were considered as one class to train a binary classifier of B and T cells (Fig. 4a). The accuracy of the optimized classifier was 93.15% and 89.81% for the training and test cases, respectively (selected features: surface area (RI threshold = 1.342, 1.368), volume (1.368), sphericity (1.342, 1.368), protein density (1.368), and dry mass (1.342, 1.368)). Second, the CD4+ and CD8+ T cells were statistically classified also in a binary fashion (Fig. 4b). The accuracy was 87.41% and 84.38% for the training and test sets, respectively (selected features: surface area (1.342, 1.362) and sphericity (1.324, 1.362)). Lastly, the multiclass cell type classifier of the three lymphocyte cell types was constructed. The identification accuracy for the training and test were 80.65% and 75.93%, respectively (selected features: surface area (1.340), sphericity (1.370), protein density (1.370), and dry mass (1.370)). The small differences in accuracy (i.e. negligible overfitting) suggest that the trained cell type classifiers make use of the general characteristics of each lymphocyte cell type; thus the trained classification models would accurately identify the newly observed individual lymphocytes.

**Table 1:** Detailed performance of the B and T lymphocyte cell type classifiers.

Training					Test				
		Output cell type (number of cell)		Sensitivity (%)			Output cell type (number of cell)		Sensitivity (%)
		B cell	T cell				B cell	T cell	
Input cell type	B cell	96	8	92.31	Input cell type	B cell	42	3	93.33
	T cell	9	135	93.75		T cell	8	55	81.30
Specificity (%)		93.75	92.31	93.15 (overall)	Specificity (%)		81.30	93.33	89.81 (overall)

The identification accuracy and its sub-parameters, sensitivity and specificity, were also presented.

**Table 2:** Detailed performance of the CD4+ and CD8+ T lymphocyte cell type classifiers.

Training					Test				
		Output cell type (number of cell)		Sensitivity (%)			Output cell type (number of cell)		Sensitivity (%)
		CD4+ T cell	CD8+ T cell				CD4+ T cell	CD8+ T cell	
Input cell type	CD4+ T cell	54	12	81.82	Input cell type	CD4+ T cell	25	4	86.21
	CD8+ T cell	6	71	92.21		CD8+ T cell	6	29	82.86
Specificity (%)		92.21	81.82	87.41 (overall)	Specificity (%)		82.86	86.21	84.38 (overall)

The identification accuracy and its sub-parameters, sensitivity and specificity, were also presented.

**Table 3:** Detailed performance of the three lymphocyte cell types classifiers.

Training						Test					
		Output cell type (number of cell)			Sensitivity (%)			Output cell type (number of cell)			Sensitivity (%)
		B cell	CD4+ T cell	CD8+ T cell				B cell	CD4+ T cell	CD8+ T cell	
Input cell type	B cell	89	6	9	85.58	Input cell type	B cell	39	1	5	86.67
	CD4+ T cell	8	49	9	74.24		CD4+ T cell	2	21	6	72.41
	CD8+ T cell	7	9	62	79.49		CD8+ T cell	2	10	22	64.71
Specificity (%)		89.58	91.76	89.41	80.65 (overall)	Specificity (%)		93.65	86.08	89.41	75.93 (overall)

The identification accuracy and its sub-parameters, sensitivity and specificity, were also presented.

## Discussion

We demonstrated the identification of non-activated lymphocyte cell types at the single-cell level using ODT and machine learning. ODT provides quantitative morphological and biochemical information on the individual lymphocytes by measuring their 3-D RI distribution. We extracted the quantitative parameters from the 3-D tomograms and observed significant differences between the cell types at the population level. However, we failed to construct accurate cell type classifiers at the single-cell level, mainly due to the high dimensionality of the feature space and the cell-to-cell variations. To overcome this limitation, the *k*-NN (*k* = 4) algorithm was employed as a statistical classification method to systematically extract and exploit the cell-type-specific fingerprints encoded in the 3-D RI tomograms. The optimized cell type classifiers can discriminate B and T cells with high accuracy of approximately 90%. The CD4+ and CD8+ T cells could be distinguished as well with an overall accuracy of over 80%. In addition, the simultaneous multiclass identification of the three lymphocyte cell types presented an overall accuracy of over 75%.

The identification performance shows that the classification models discriminate B and T lymphocytes more precisely compared to the T cell subtype identification (CD4+ and CD8+ T cells); these results imply that the differences in cellular morphology and biochemical properties between the B and T cells are more distinct than those between the CD4+ and CD8+ T cells. This observation is consistent with the previous knowledge of the lymphocyte-differentiation pathway^54^. The B and T lymphocytes originate from hematopoietic stem cells and then mature in different organs. Thus, the lymphocytes have similar cellular phenotypes such as one large nucleus and spherical shapes; however, the B and T cells have entirely different cellular functions. Even though our method established the cell type classifiers by optimizing the features based on the statistical performance instead of biological relevance, the machine learning algorithm automatically recognizes and exploits the distinct differences in the morphological and biochemical properties between the lymphocyte cell types.

The present approach combines 3-D RI tomography and statistical classification, for the first time to our knowledge, which provides several advantages. First, the present method enables the identification of lymphocyte cell types exploiting intrinsic optical properties of cells, which cannot be achieved by conventional optical microscopic techniques without using fluorescent methods. As ODT measures intrinsic 3-D RI distribution of individual lymphocytes, it provides consistent and highly reproducible results. In contrast, labeling methods such as fluorescence microscopy techniques can provide molecular-specific localization information. However, their signals are generally qualitative, and may vary significantly depending on experimental protocols, skills, and equipment. This variability of labeling methods may decrease overall identification accuracy. Alternatively, existing bright-field or phase contrast microscopy can be used to measure 2-D morphological features, such as projected area, aspect ratio, and nucleus size. However, these qualitative image data obtained with these conventional bright-field or phase contrast microscopy only provide limited information. We found that statistical classification performed by morphological information only (surface area, cellular volume, and sphericity) lowers overall accuracy (Supplementary Fig. 3). This result clearly indicates the advantages of using the 3-D RI tomogram. Second, the present method uses a simple and cost-effective optical setup compared to fluorescence-activated cell sorting or other label-free techniques such as Raman spectroscopy. Recently, a 3-D holographic microscope has become commercially available, which simplifies the optical system and achieves over 100 tomograms per second by exploiting a digital micromirror device to reduce the required time for measuring a 3-D RI tomogram^55, 56^. Thus, the present approach can be easily transferred to basic research facilities and clinics. Lastly, there is no limitation in applying the present method to discriminate other types of cells including RBCs, cancer cells, neurons, and glial cells. Because ODT has been widely used to measure various biological samples, the present approach can be readily used to identify various cell types.

One of the limitations of the current study is that antibody-labelled lymphocytes were used to demonstrate the proof of principle. Because labeling and sorting procedures are inevitable to confirm the presence of antibodies in lymphocytes, and the use of antibody labeling is a standard technique to assure the subtypes of antibody-specific classification. Nonetheless, this approach still uses the intrinsic imaging contrast – 3-D RI tomography, and the labeling agents used to specify antibodies have negligible effects to the measured 3-D RI signal. CD4 antibody bound per cell value is about 48,000 and the total number of protein per cell of a eukaryotic cell is in the order of billions^57, 58^. Thus, antibody staining accounts for less than 0.0005% of total protein numbers, which can be ignored in the measured 3-D RI tomogram because the RI signals attribute to total protein distributions.

From the algorithmic point of view, there are several points to be improved for practical use of the proposed technique. As described earlier, we compared several statistical classification algorithms including the *k*-NN (*k* = 4 and *k* = 6), linear discrimination analysis, quadratic discrimination analysis, naïve Bayes, and decision tree (Supplementary Fig. 2), and selected the *k*-NN (*k* = 4) as the classification model. While we tested several machine learning algorithms exploiting quantitative features with different thresholds widely used in ODT-based studies, employing advanced features extraction methods and statistical classification models could enhance the overall identification performance. Unfortunately, designing powerful features for 3-D biological microscopy data, especially for 3-D RI tomograms, has been largely unexplored and is beyond the scope of this proof-of-concept study. The methods developed in different disciplines could be translated to facilitate this direction: scale-invariant feature transform for X-ray computerized tomography^59^ or histogram-based features for lidar-based point clouds^60^. However, these ‘shallow’ descriptors require laborious optimization procedures specific to samples and imaging setups employed.

We expect that ‘deep’ learning, a state-of-the-art machine learning technique based on multilayered neural networks, could be a powerful and generic feature extraction strategy for 3-D biological microscopy. Recently, our group successfully combined 2-D holographic microscopy and deep learning for the label-free screening of multiple pathogens^61^. Upon extension to 3-D ODT, the remarkable flexibility and learning abilities of deep neural networks would let us fully exploit the complex information encoded in the 3-D RI tomograms and dramatically enhance the identification performance. An important step in this direction is to combine the proposed method with high-throughput imaging technologies^62^ to obtain a large size of training data sufficient for deep learning.

In summary, we envision that ODT combined with machine learning will be a useful tool in biomedical research. ODT quantitatively provides the morphological and biochemical characteristics of the samples, and then machine learning enables the label-free identification of cell types using the measured quantitative information. The present method can be widely used in the study of immunology, cancer biology, and neuroscience.

## Methods

### Mice

C57BL/6 J mice (gender and age-matched, 6–8 weeks) were purchased from Daehan Biolink (Republic of Korea). Animal care and experimental procedures were performed under approval of the Institutional Animal Care and Use Committee of KAIST (KA2010-21, KA2014-01 and KA2015-03). All the experiments in this study were carried out in accordance with the approved guidelines.

### Flow cytometry for lymphocyte sorting

White blood cells were isolated from the blood harvested from the heart of mice. Erythrocytes were removed by ACK lysis. Cells were blocked with anti-CD16/32 and then stained for surface molecules. DAPI (4,6-diamidino-2-phenylindole; Roche, Switzerland) was used for dead cell exclusion. Sorting was performed on an Aria II or III system (BD Biosciences, CA) using an 85-μm nozzle or Astrios system (Beckman Coulter, CA) using a 70-μm nozzle. Antibodies for flow cytometry were purchased from BD Biosciences, eBioscience (CA), Biolegend (CA). The antibodies used were CD3ε (clone 17A2), CD4 (GK1.5), CD8α (53-6.7), CD19 (1D3), CD45R (B220, RA3-6B2), NK1.1 (PK136).

### 3-D refractive index tomography

To reconstruct the 3-D RI tomograms of lymphocytes, a Mach-Zehnder interferometric microscope was used^15^ (Fig. 1d). A laser beam from a diode-pumped solid-state laser (*λ* = 532 nm, 100 mW, Shanghai Dream Laser Co., China) is split into two arms using a beam splitter. One arm illuminates a sample with various illumination angles ranging from −60° to 60° in air at the sample plane with respect to the optic axis, which is systematically controlled with a dual-axis galvanomirror (GVS012, Thorlabs, NJ), and the other is used as a reference beam. The sample is placed between a condenser lens (UPLSAPO Water 60×, numerical aperture (NA) = 1.2, Olympus, Japan) and an objective lens (PLAPON Oil 100×, NA = 1.4, Olympus, Japan). For a single cell level analysis, we locate a single cell in the field of view using a manual translation stage. The diffracted light from the sample is then collected by the objective lens and projected onto the camera plane. At the camera plane, the sample beam interferes with the reference beam, generating spatially modulated holograms, which are then captured by a CMOS camera (1024 PCI, Photron USA Inc., CA). For reconstructing a 3-D RI tomogram, a total of 300 holograms of a sample are measured with a frame rate of 1000 Hz by changing the angle of illuminations which takes 0.3 s. Then, the optical field information (amplitude and phase) of the measured holograms are retrieved using a field retrieval algorithm based on Fourier transform^49, 50^. From the retrieved multiple amplitude and phase information, a 3-D RI tomogram is reconstructed using an optical diffraction tomography algorithm^14, 51^. An iterative regularization algorithm with a non-negativity constraint was used to fill the missing cone information which results from the limited NA of the condenser and objective lenses^63^. Details on reconstructing 3-D RI tomograms can be found elsewhere^15, 64^. The experimental resolution of our setup estimated by imaging a micro-bead was 373 nm and 496 nm for lateral and axial directions, respectively, which is consistent with the theoretical values^65^.

### Image processing and statistical analysis

Image processing was performed with Matlab (R2014b; MathWorks Inc., MA) and ImageJ (the National Institutes of Health, MD). The RI isosurfaces were rendered with commercial software (TomoStudio^TM^, Tomocube Inc., Republic of Korea). Statistical analysis was done with GraphPad Prism (GraphPad Software Inc., CA). *P*-values were calculated by Student’s *t*−test.

### Calculation of the quantitative structural and biochemical characteristics

The quantitative structural and biochemical parameters of the individual lymphocytes were calculated from the measured 3-D RI tomograms. To calculate the cellular surface area *S* and volume *V*, the voxels with the RI values higher than the threshold RI value were selected for segmentation from a 3-D RI tomogram of a lymphocyte. The surface are and volume were calculated from the number of voxels at the boundary and inner region of the segmented region, respectively. The sphericity, which is a dimensionless parameter that indicates the roundness of a lymphocyte, was obtained from the calculated surface area and volume as follows: *Sphericity* = *π* ^*1/3*^·(*6* *V*)^*2/3*^/*S*. The biochemical characteristics (protein density and dry mass) were obtained from the RI values due to the well-characterized linear relation between the RI value and the local concentration of non-aqueous molecules (i.e., proteins, lipids, and nucleic acids inside cells; mostly proteins). RI values were converted to the protein density *C* with the following relation: *n* = *n*_*0*_ + *αC*, where *n* and *n*_*0*_ are the RI values of a voxel and the medium, respectively, and *α* is the refractive index increment (RII). Because it is known that most proteins have similar RII values, we used a RII value of 0.2 mL/g in this study. The total dry mass of a lymphocyte was calculated by simply integrating the protein density over the cellular volume. Details on calculating the quantitative information from 3-D RI tomograms can be found elsewhere^23, 25^.

### Machine learning

We investigated the 100-dimensional feature space as described in the main text. We selected the *k*-NN (*k* = 4) as the classification model after comparing several algorithms (Supplementary Fig. 2). The *k*-NN algorithm predicts the class of a newly observed data by choosing the most frequent class labels of *k* nearest neighbour data points in the feature space. We standardized all features prior training and test because *k*-NN is sensitive to pre-processing. Since there exists substantial redundancy between the features and it is desirable to choose minimal number of features to reduce overfitting, it was crucial to select the optimal feature set. We exhaustively searched all combinations of the morphological and biochemical features obtained at a single or two different RI threshold values. The feature set with the highest cross-validation accuracy was selected. The optimized classifier was tested using the data that was not utilized for training.

## Electronic supplementary material

Video 1

Video 2

Video 3

Supplementary information
