# Supplementary material for: Identification of non-activated lymphocytes using three-dimensional refractive index tomography and machine learning
Source: Sci Rep. 2017 Jul 27;7:6654. doi: 10.1038/s41598-017-06311-y (PMC5532204; doi:10.1038/s41598-017-06311-y)
Supplement: Supplementary file 4 — Supplementary information [file 41598_2017_6311_MOESM4_ESM.pdf]

Supplementary Information

Identification of non-activated lymphocytes using three-dimensional refractive index tomography and machine learning

Jonghee Yoon[1,2,+,†] , YoungJu Jo[1,2,+], Min-hyeok Kim[3], Kyoohyun Kim[1,2], SangYun Lee[1,2], Suk-Jo Kang[3], and YongKeun Park[1,2,4,*]

[1]*Department of Physics, Korea Advanced Institute of Science and Technology (KAIST), Daejeon 34141, Republic of Korea*

[2]*KAIST Institute Health Science and Technology, Daejeon 34141, Republic of Korea*

[3]*Department of Biological Sciences, KAIST, Daejeon 34141, Republic of Korea*

[4]*Tomocube, Inc., Daejeon 34051, Republic of Korea*

[+]*These authors contributed equally to this work.*

[†] *Present address: Department of Physics, University of Cambridge, Cambridge, CB3 0HE, UK.*

Correspondence:

YongKeun Park, Ph.D.

KAIST, 291 Daehak-ro, Yuseong-gu, Daejeon 34141, Republic of Korea

Phone: +82-42-350-2514; Fax: +82-42-350-2510

Email: yk.park@kaist.ac.kr

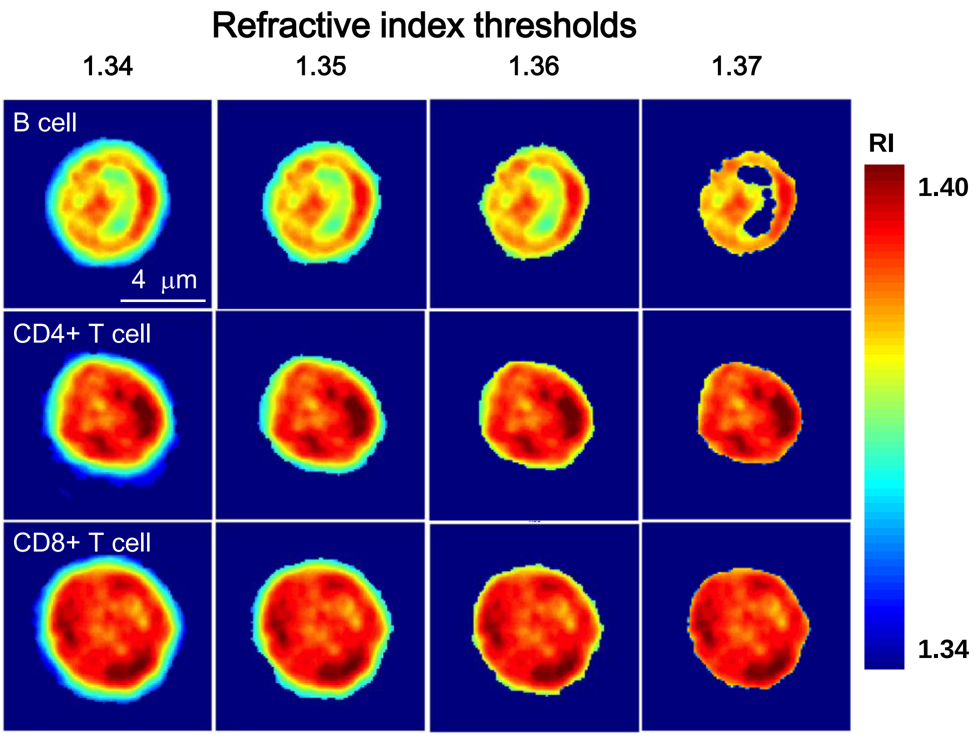

Supplementary Figure 1. Representative x-y slices of 3-D RI tomograms of the three lymphocyte cell types. Each slice shows RI distribution which is higher than RI threshold. Note that lower threshold RI values present the overall morphology, while higher threshold RI values tend to reveal information on the intracellular components. Scale bar, 4 µm.

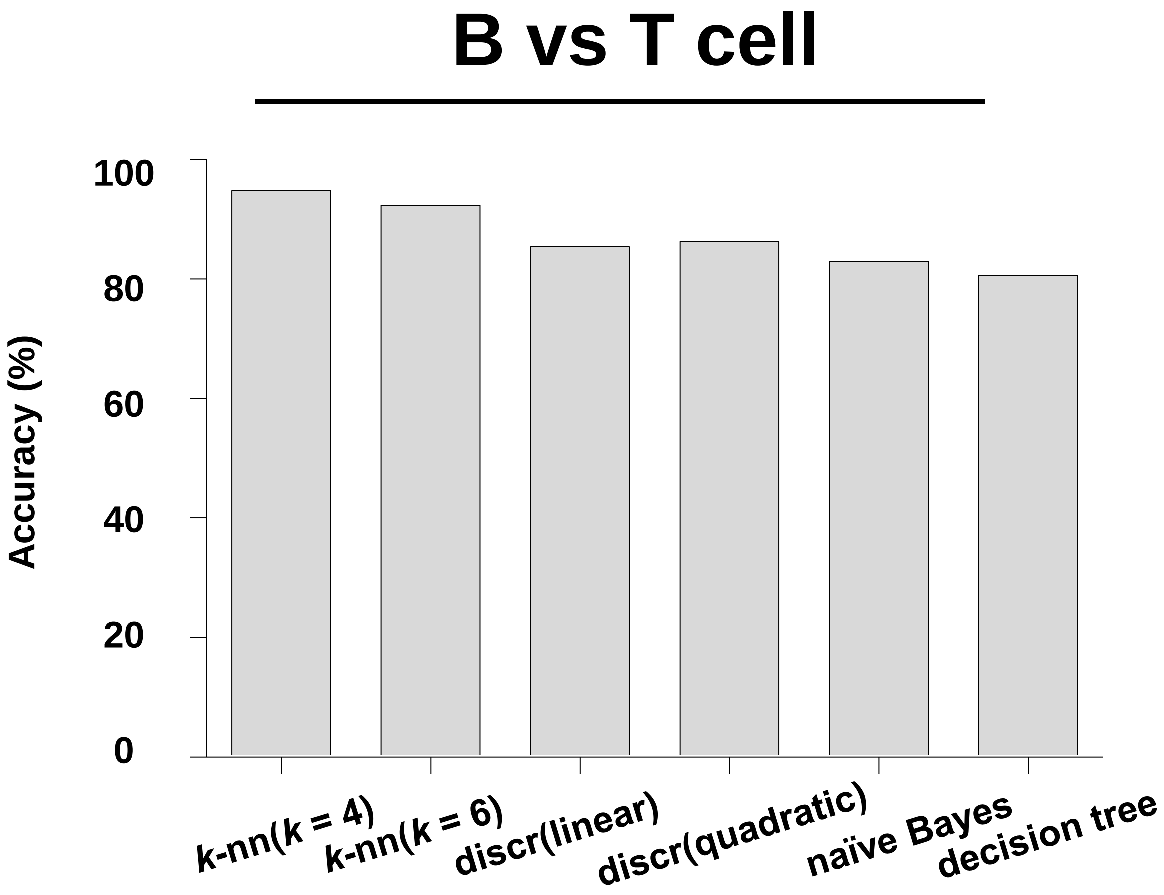

Supplementary Figure 2. Comparison of several statistical classification models (*k*-NN (*k* = 4 and *k* = 6), linear discrimination analysis, quadratic discrimination analysis, naïve Bayes, and decision tree).

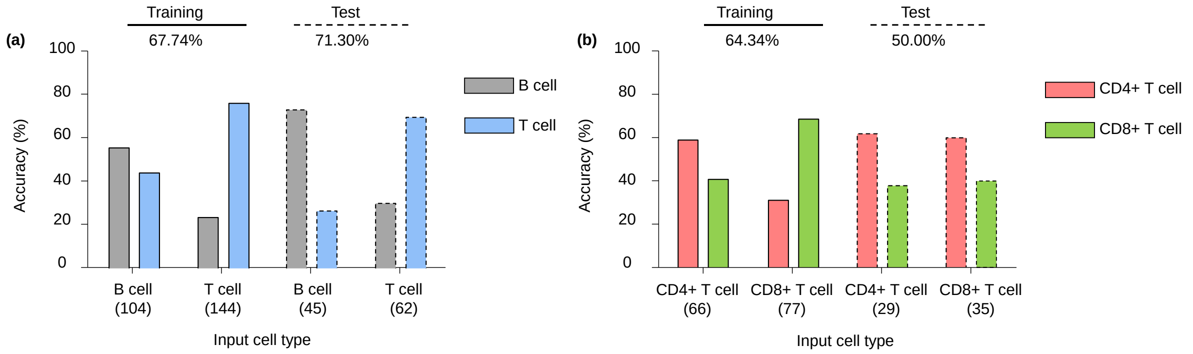

Supplementary Figure 3. Identification performance of the optimized lymphocyte cell type classifiers using morphological features (surface area, cellular volume, and sphericity) only. (a) Binary classification of B and T cells. (b) Binary classification of CD4+ and CD8+ T cells. The numbers below the cell types indicate the number of cells used.

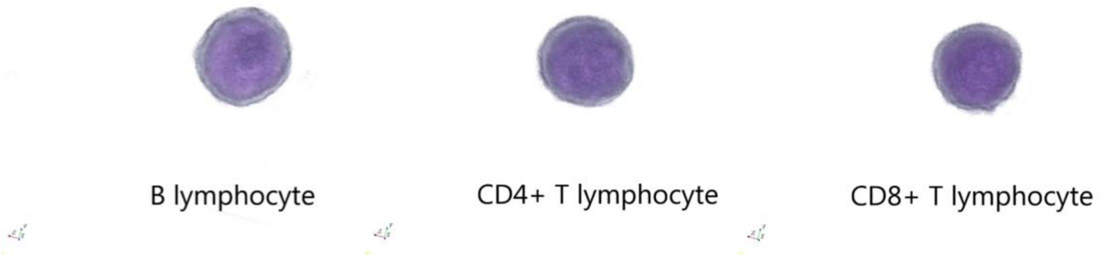

Supplementary Video 1-3. 3-D rendered lymphocyte tomograms (B cell, CD4+ T cell, and CD8+ T cell)
